# Male circumcision, religion, and infectious diseases: an ecologic analysis of 118 developing countries

**DOI:** 10.1186/1471-2334-6-172

**Published:** 2006-11-30

**Authors:** Paul K Drain, Daniel T Halperin, James P Hughes, Jeffrey D Klausner, Robert C Bailey

**Affiliations:** 1 University of Washington School of Medicine, Seattle, USA; 2 United States Agency for International Development, Southern Africa Regional HIV-AIDS Program, Mbabane, Swaziland; 3 Center for AIDS and STD, University of Washington, Seattle, USA; 4 San Francisco Department of Public Health, San Francisco, USA; 5 Division of Epidemiology, University of Illinois at Chicago, Chicago, USA

## Abstract

**Background:**

Both religious practices and male circumcision (MC) have been associated with HIV and other sexually-transmitted infectious diseases. Most studies have been limited in size and have not adequately controlled for religion, so these relationships remain unclear.

**Methods:**

We evaluated relationships between MC prevalence, Muslim and Christian religion, and 7 infectious diseases using country-specific data among 118 developing countries. We used multivariate linear regression to describe associations between MC and cervical cancer incidence, and between MC and HIV prevalence among countries with primarily sexual HIV transmission.

**Results:**

Fifty-three, 14, and 51 developing countries had a high (>80%), intermediate (20–80%), and low (<20%) MC prevalence, respectively. In univariate analyses, MC was associated with lower HIV prevalence and lower cervical cancer incidence, but not with HSV-2, syphilis, nor, as expected, with Hepatitis C, tuberculosis, or malaria. In multivariate analysis after stratifying the countries by religious groups, each categorical increase of MC prevalence was associated with a 3.65/100,000 women (95% CI 0.54-6.76, p = 0.02) decrease in annual cervical cancer incidence, and a 1.84-fold (95% CI 1.36-2.48, p < 0.001) decrease in the adult HIV prevalence among sub-Saharan African countries. In separate multivariate analyses among non-sub-Saharan African countries controlling for religion, higher MC prevalence was associated with a 8.94-fold (95% CI 4.30-18.60) decrease in the adult HIV prevalence among countries with primarily heterosexual HIV transmission, but not, as expected, among countries with primarily homosexual or injection drug use HIV transmission (p = 0.35).

**Conclusion:**

Male circumcision was significantly associated with lower cervical cancer incidence and lower HIV prevalence in sub-Saharan Africa, independent of Muslim and Christian religion. As predicted, male circumcision was also strongly associated with lower HIV prevalence among countries with primarily heterosexual HIV transmission, but not among countries with primarily homosexual or injection drug use HIV transmission. These findings strengthen the reported biological link between MC and some sexually transmitted infectious diseases, including HIV and cervical cancer.

## Background

Geographical variations in HIV prevalence have been observed between less-developed and more-developed countries, as well as within regions of similar socioeconomic development [1-5]. The epidemiology of HIV and other infectious diseases have been associated with both religious practices and male circumcision [1-19]. Religious beliefs and practices dictate many societal and sexual behaviors that influence transmission of sexually-transmitted infections (STIs) [20]. Male circumcision has been more common among populations with lower rates of HIV, cervical cancer, and other STIs [3,10-13], and shown in one randomized trial to reduce HIV transmission [21]. Although religious affiliation is a major determinant of male circumcision status [12], many analyses have not controlled for religion when examining relationships between male circumcision and infectious diseases.

This study builds upon and further expands our previously reported analyses of variables associated with country-specific HIV prevalence and cervical cancer incidence [5,22]. In our extensive analysis of HIV co-factors, among 81 variables male circumcision had the strongest association with HIV prevalence [5]. We now present a more thorough examination of the association between male circumcision and HIV prevalence by better adjusting for religion, by separately analyzing the sub-Saharan African region, and by conducting separate analyses between countries with sexual versus non-sexual primary modes of HIV transmission. Our previous ecological analysis of cervical cancer utilized 54 country-level variables, but did not include the important determinant of male circumcision [13,22]. We complete our previous analysis by describing the relationships between male circumcision and cervical cancer, and by including male circumcision in the previously reported multivariate model. In addition, we further expand on our previous studies by describing the epidemiology of male circumcision among developing countries and by describing associations between male circumcision and five other infectious diseases.

## Methods

We conducted an ecological study of country-level variables among developing countries. The United Nations Development Programme (UNDP) 'Human Development Report 2004' provided the Human Development Index, which determines each country's development status on the basis of life expectancy, educational attainment, and adjusted real income [23]. Countries classified as high human development ("developed") countries were excluded from the analyses under the assumption that they have greater capacity to sustain national treatment and prevention programs, and have different epidemiological infectious disease profiles. Therefore, subsequent country-specific data were collected for 122 low and medium human development ("developing") countries.

### Data collection

The Joint United Nations Programme on HIV/AIDS (UNAIDS) provided country-specific age-standardized HIV seroprevalence per 100 adults 15–49 years old for the year 2004 for 100 countries [24]. Countries with <0.1% of adults infected with HIV were considered to have an adult seroprevalence of 0.05%. The International Agency for Research on Cancer provided country-specific annual age-standardized cervical cancer incidence [a surrogate measure for Human Papillomavirus (HPV)] per 100,000 females for the year 2000 for 117 countries and the most recent country-specific Herpes Simplex Virus type-2 (HSV-2) seroprevalence per 100 women for 23 countries [25,26]. The World Health Organization provided the most recent country-specific syphilis seroprevalence per 10,000 women for 43 countries [27], and Hepatitis C prevalence per 100 adults for 75 countries [28]. The UNDP provided country-specific prevalence of all forms of tuberculosis per 100,000 people for the year 2002 for 110 countries and malaria prevalence per 10,000 people for the year 2000 for 94 countries [23]. We did not include chlamydia or gonorrhea in this analysis due to a lack of available data.

We used survey data from various published sources [3,11-13,15,29,30], including Demographic and Health Surveys and Behavioral Surveillance Surveys [31-35] to categorize the country-wide prevalence of male circumcision as "low" (<20%), "intermediate" (20–80%), or "high" (>80%), as we have previously done [5,10]. These categories were chosen to best minimize misclassification. When published country-specific data were not available, we used ethnographic confidence methods, as previously utilized by Halperin and Bailey [5,10], to categorize country-specific male circumcision prevalence as low, intermediate, or high. The classification of country-specific male circumcision prevalence was independently verified by three colleagues (listed in Acknowledgements). We omitted four developing countries (Armenia, Belarus, Kyrgyzstan, and Vanuatu) from all analyses because their male circumcision prevalence could not be confidently categorized.

The United Nations Population Division provided all population statistics estimated for the year 2000 [36]. UNAIDS provided data for geographical regions [24]. We used the first listed mode of transmission from the UNAIDS 'HIV/AIDS Epidemic Update 2002' as the primary mode of HIV transmission for each geographical region [37]. The United States Central Intelligence Agency 'The World Factbook' provided both the percentage of Muslims and the percentage of Christians within each country [38].

### Statistical analyses

Statistical analyses were conducted on 118 developing countries using Stata Version 8.0 [39]. All regression statistics were performed using a robust variance to account for unmeasured ecologic and population differences. We conducted separate analyses of HIV prevalence among sub-Saharan African and non-sub-Saharan African countries due to the differing severity and nature of the epidemics [40-42]. We also conducted separate analyses among non-sub-Saharan African regions whose primary mode of HIV transmission was heterosexual contact versus homosexual contact or injection drug use [24]. Heterosexual contact was the primary mode of HIV transmission for the sub-Saharan African region. HIV seroprevalence was natural log (ln)-transformed to create a more normal distribution for regression analyses. Countries were categorized into tertiles as having low (<5%), intermediate (5–55%), and high (>55%) percentage of predominantly Muslims, and as having low (<6%), intermediate (6–55%), and high (>55%) percentage of predominantly Christians.

The mean and standard deviation for each infectious disease were separately summarized among countries with low and high male circumcision prevalence. Means and standard deviations for cervical cancer incidence and HIV prevalence were also separately summarized among countries with low and high male circumcision prevalence within each religious tertile group. Means and standard deviations were analyzed for statistical significance between high versus low male circumcision groups using 2-sample *t *tests for independent samples with unequal variances.

Univariate linear regression statistics examined category of male circumcision prevalence with each infectious disease. Results are presented by order of the *R*^2 ^value, which estimates the amount of the variance explained by the association. Since only HIV prevalence and cervical cancer incidence were significantly associated with male circumcision in univariate analyses, subsequent multivariate analyses were only performed for these two outcomes. Multivariate analyses of covariance models included each country's percentage of the population Muslim and percentage of the population Christian.

Additional multivariate analyses were conducted for cervical cancer incidence and HIV prevalence based on our previously published models [5,22]. In brief, a multivariate model of cervical cancer incidence was conducted adjusting for the 6 additional variables that were previously found to be significant (p < 0.05) indicators of cervical cancer incidence. Definitions and sources of these variables (number of doctors per 100,000 people, percent of children immunized for measles, female disability-adjusted life expectancy in years, percent of female adult illiteracy rate, percent of infants with low birth weight, and geographic region as defined by the International Agency for Research on Cancer), have been previously described [22]. Similarly, a multivariate model of HIV prevalence was conducted adjusting for the 6 additional variables that were previously found to be significant (p < 0.05) indicators of HIV prevalence. Detailed methods, including definitions and sources of these variables (years since HIV was first reported, geographic region, percent of the population younger than 25 years, percent of female adult illiteracy rate, percent of children fully immunized for diphtheria, tetanus, and pertussis, and number of doctors per 100,000 people), have been previously described [5].

## Results

### Male circumcision prevalence

The classification of male circumcision prevalence is listed in Table 1 for 118 developing countries. Among these, 53 countries, containing 700 million males, were categorized as having a high (>80%) male circumcision prevalence, 14 countries, containing 135 million males, were categorized as having an intermediate (20–80%) male circumcision prevalence, and 51 countries, containing 1.6 billion males, were categorized as having a low (<20%) male circumcision prevalence.

**Table 1:** Category of male circumcision prevalence for 118 developing countries.

**Male Circumcision Prevalence**				
Low (<20%)		Intermediate (20–80%)	High (>80%)	
		
Belize	Micronesia, Fed States	Albania	Afghanistan	Liberia
Bhutan	Moldova, Rep of	Bosnia Herzegovina	Algeria	Libyan Arab Jama
Bolivia	Mongolia	Central African Republic	Angola	Madagascar
Botswana	Myanmar	Cote d'Ivoire	Azerbaijan	Malaysia
Brazil	Namibia	Ethiopia	Bangladesh	Maldives
Bulgaria	Nepal	Kazakhstan	Benin	Mali
Burundi	Nicaragua	Lesotho	Burkina Faso	Mauritania
Cambodia	Panama	Macedonia, FYR of	Cameroon	Mauritius
Cape Verde	Papua New Guinea	Mozambique	Chad	Morocco
China	Paraguay	South Africa	Comoros	Niger
Colombia	Peru	Sudan	Congo (Brazzaville)	Nigeria
Dominican Republic	Romania	Tanzania, United Rep of	Dem Rep of the Congo	Oman
Ecuador	Russian Federation	Uganda	Djibouti	Pakistan
El Salvador	Rwanda	Yugoslavia	Egypt	Philippines
Fiji	Samoa		Equatorial Guinea	Saudi Arabia
French Polynesia	Solomon Islands		Eritrea	Senegal
Georgia	Sri Lanka		Gabon	Sierra Leone
Guatemala	Suriname		Gambia	Somalia
Guyana	Swaziland		Ghana	Syrian Arab Rep
Haiti	Thailand		Guinea	Tajikistan
Honduras	Ukraine		Guinea-Bissau	Togo
India	Venezuela		Indonesia	Tunisia
Jamaica	Viet Nam		Iran, Islam Rep of	Turkey
Korea, DPR	Zambia		Iraq	Turkmenistan
Lao, PDR	Zimbabwe		Jordan	Uzbekistan
Malawi			Kenya	Yemen
			Lebanon	

Male circumcision prevalence had a distinct geographical pattern. Thirteen of 14 (93%) developing countries in North Africa and the Middle East had a high male circumcision prevalence. Twenty-eight of 45 (62%) sub-Saharan African countries had a high male circumcision prevalence. Eight of 27 (30%) Southeast Asian and Pacific Island countries had a high male circumcision prevalence, and most circumcised males resided in Indonesia, Pakistan, Bangladesh, or the Philippines. Only 4 of 18 (22%) developing countries in Europe and Central Asia had a high male circumcision prevalence, and all 18 developing countries in Latin American and the Caribbean region had a low male circumcision prevalence.

### Male circumcision and religion

As expected, male circumcision was strongly associated with religious variables (data not presented). A greater percent of the population being Muslim was strongly associated with more male circumcision prevalence (p < 0.001). Conversely, a greater percent of the population being Christian was strongly associated with less male circumcision (p < 0.001). Among 49 countries with high male circumcision prevalence, the mean percentage of the population Muslim was 69% and the mean percentage of the population Christian was 16%.

### Male circumcision and infectious diseases

In univariate regression analyses, male circumcision was associated with HIV prevalence among sub-Saharan African countries, HIV prevalence among non-sub-Saharan African countries with primarily heterosexual contact, HIV prevalence among non-sub-Saharan countries with primarily homosexual contact or injection drug use, and cervical cancer incidence (Table 2). Male circumcision was not associated with HSV-2, syphilis, nor, as expected, with the non-sexually-transmitted-disease prevalences of Hepatitis C, tuberculosis, or malaria. Among sub-Saharan African countries, HIV prevalence was 3.0% among countries with a high male circumcision prevalence and 16.5% among countries with a low male circumcision prevalence (p < 0.001). Among non-sub-Saharan African countries with primarily heterosexual HIV transmission, HIV prevalence was 0.09% among countries with a high male circumcision prevalence and 0.76% among countries with a low male circumcision prevalence (p < 0.001). Similarly, the mean annual cervical cancer incidence was 20.5/100,000 women among countries with a high male circumcision prevalence and 35.0/100,000 women among countries with a low male circumcision prevalence (p < 0.001). Although there was no significant association between male circumcision and HSV-2 prevalence, the size and direction of the coefficient suggests that male circumcision could be associated with reduced HSV-2 prevalence given a larger sample size.

**Table 2:** Male circumcision prevalence and selected infectious diseases among developing countries.

	Univariate linear regression of male circumcision prevalence^1^			Countries with low (<20%) male circumcision prevalence		Countries with high (>80%) male circumcision prevalence	
			
	No. of countries	Regression coefficient	*R*^2^*	No. of countries	Mean ± SD*	No. of countries	Mean ± SD*
	
HIV prevalence among sub-Saharan Africa (/100 adults)^2^	38	-0.90	**0.51**	8	**16.48 ± 0.002**	22	**2.98 ± 0.002**
HIV prevalence among non-sub-Saharan African countries with primarily heterosexual HIV transmission (/100 adults)^2^	29	-1.08	**0.51**	11	**0.76 ± 0.004**	17	**0.09 ± 0.002**
HIV prevalence among non-sub-Saharan African countries with primarily homosexual or injection drug use HIV transmission (/100 adults)^2^	33	-1.03	**0.29**	25	*0.41 *± *0.004*	4	*0.06 *± *0.001*
Cervical cancer incidence (/100,000 women/year)	117	-7.2	**0.18**	51	**35.0 ± 16.2**	52	**20.5 ± 12.8**
							
Herpes Simplex Virus type-2 prevalence (/100 women)	23	-6.1	0.08	10	42.9 ± 13.8	9	30.2 ± 21.6
Tuberculosis prevalence (/100,000)	110	25.9	0.01	48	244 ± 191	49	296 ± 244
Hepatitis C prevalence (/100 adults)	75	0.20	0.003	37	3.17 ± 3.90	34	3.46 ± 3.81
Syphilis prevalence (/10,000 women)	43	51.7	0.0006	20	295 ± 235	14	284 ± 317
Malaria prevalence (/10,000)	94	-31.7	0.0007	31	385 ± 887	31	331 ± 596

SD – standard deviation.

* R^2 ^values and mean values in bold type had p-values <0.001 and in italics type had p-values <0.05. R^2 ^values and mean values not in bold or italics type had p-values >0.05.

^1 ^Male circumcision prevalence was coded as 1 = low (<20%), 2 = intermediate (20–80%), and 3 = high (>80%).

^2 ^Regression analyses presented as natural log of HIV (prevalence/100,000 adults).

### Religion and infectious diseases

In univariate regression analyses, religion was also strongly associated with HIV and cervical cancer. Each percent increase of the population Muslim was associated with a 0.21/100,000 women (CI 0.15-0.28, p < 0.001) decrease in cervical cancer incidence, and a 1.25-fold (CI 1.16-1.35, p < 0.001) and 1.19-fold (CI 1.12-1.27, p < 0.001) decrease in adult HIV prevalence among sub-Saharan African and non-sub-Saharan African countries, respectively. Similarly, each percent increase of the population Christian was associated with a 0.26/100,000 women (CI 0.19-0.34, p < 0.001) increase in cervical cancer incidence, and a 1.32-fold (CI 1.20-1.45, p < 0.001) and 1.20-fold (CI 1.12-1.30, p < 0.001) increase in adult HIV prevalence among sub-Saharan African and non-sub-Saharan African countries, respectively.

### Male circumcision, religion, and infectious diseases

**Figure 1:**
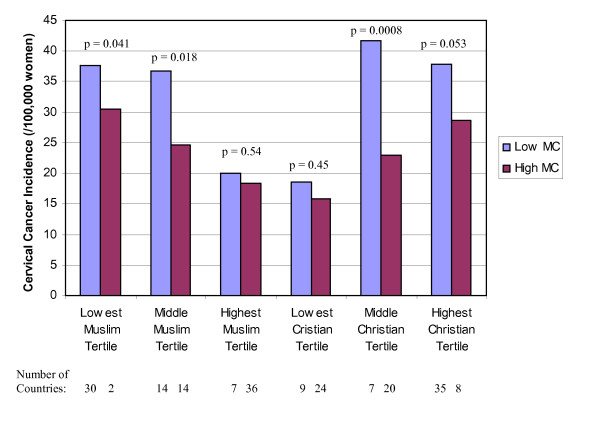
Cervical cancer incidence (/100,000 women) by low (<20%) and high (>80%) male circumcision (MC) prevalence and tertiles of the percent Muslim and Christian among 121 developing countries.

**Figure 2:**
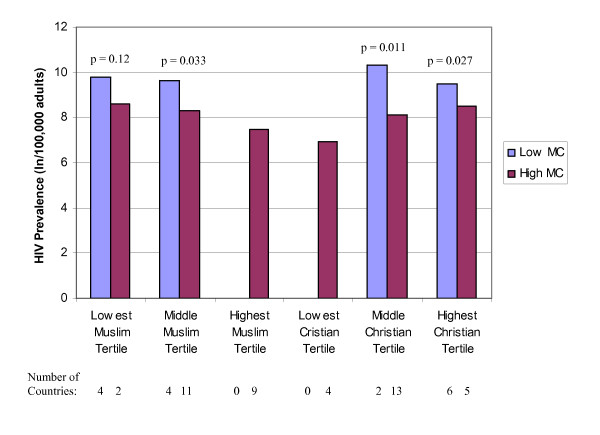
Natural log HIV prevalence (/100,000 adults) by low (<20%) and high (>80%) male circumcision (MC) prevalence and tertiles of the percent Muslim and Christian among 38 sub-Saharan African countries with primarily heterosexual HIV transmission.

**Figure 3:**
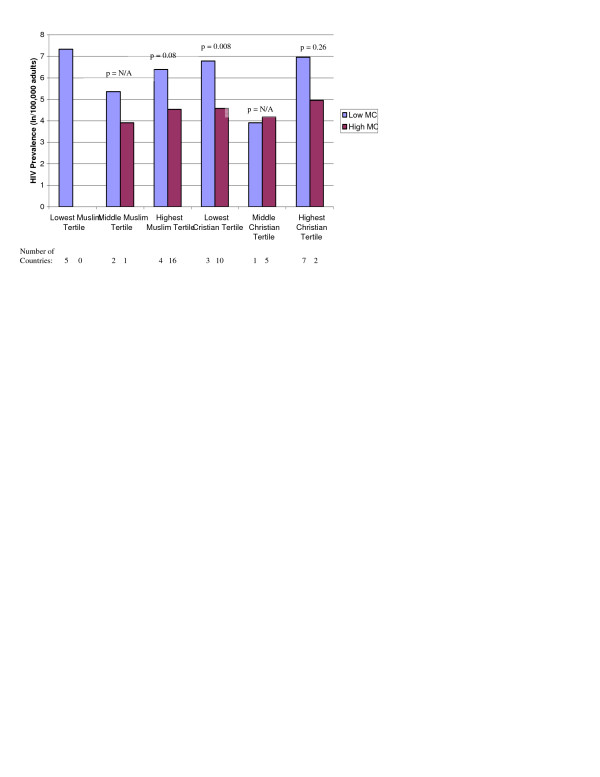
Natural log HIV prevalence (/100,000 adults) by low (<20%) and high (>80%) male circumcision (MC) prevalence and tertiles of the percent Muslim and Christian among 29 non-sub-Saharan African countries with primarily heterosexual HIV transmission.

Mean rates of cervical cancer incidence and HIV prevalence are presented among countries with low and high male circumcision prevalence in Figures 1, 2, 3, after separately stratifying countries into tertiles for percent Muslim and percent Christian. In nearly all religious tertile groups, mean cervical cancer incidence or HIV prevalence was lower among countries with a high male circumcision prevalence. Mean differences between high and low male circumcision groups were statistically significant for 7 of 14 (50%) tertile groups, and 2 additional tertile groups (14%) showed a borderline significance. When excluding each of the major religions in separate multivariate analyses, there were no meaningful changes in the associations between infectious diseases and male circumcision prevalence.

In a multivariate model, percent of the population Christian and male circumcision, but not percent of the population Muslim, remained independently associated with cervical cancer incidence (Table 3). After stratifying the countries by religious groups, each categorical increase of male circumcision prevalence was associated with a 3.65/100,000 women (CI 0.54-6.76, p = 0.02) decrease in annual cervical cancer incidence. These 3 measures (prevalence of male circumcision, percent Muslim, and percent Christian) accounted for 40% of the variance in cervical cancer incidence among the 105 developing countries included in the model. In a multivariate model adjusted for 6 additional country-specific variables previously found to be associated with cervical cancer incidence [22], each categorical increase of male circumcision prevalence was highly associated with a 10.38/100,000 women (CI 4.94-15.82, p < 0.001) decrease in annual cervical cancer incidence. The 9 variables accounted for 59% of the variance in cervical cancer incidence among the 85 countries included in the model. Furthermore, male circumcision had a stronger association with cervical cancer incidence than all the other variables included in the model.

**Table 3:** Multivariate linear regression models of cervical cancer incidence (/100,000 women/year) among developing countries.

	No. of countries	coefficient	p-value	*R*^2^
**Unadjusted regression model**	**105**			**0.40**
Percent of population Muslim		-0.010	0.84	
Percent of population Christian		0.21	<0.001	
Male circumcision prevalence^1^		-3.65	0.022	
				
**Adjusted regression model**^2^	**85**			**0.59**
Percent of population Muslim		0.083	0.25	
Percent of population Christian		0.13	0.017	
Male circumcision prevalence^1^		-10.38	<0.001	

^1 ^Male circumcision prevalence was coded as 1 = low (<20%), 2 = intermediate (20–80%), and 3 = high (>80%).

^2 ^Model adjusted by country-specific measures including number of doctors per 100,000 people, percent of children immunized for measles, female disability-adjusted life expectancy in years, percent of female adult illiteracy rate, percent of infants with low birth weight, and major geographic region.

Among sub-Saharan African countries, percent of the population Muslim and male circumcision, but not percent of the population Christian, remained independently associated with HIV prevalence (Table 4). After stratifying the countries by religious groups, each categorical increase of male circumcision prevalence was associated with a 1.84-fold (CI 1.36-2.48, p < 0.001) decrease in adult HIV prevalence. In a multivariate model adjusted for 6 additional country-specific variables previously found to be associated with HIV prevalence [5], each categorical increase of male circumcision prevalence was associated with a 2.27-fold (CI 1.51-3.42, p = 0.001) decrease in adult HIV prevalence.

**Table 4:** Multivariate linear regression models of HIV prevalence (/100,000 adults) among developing countries.^1^

	Sub-Saharan African countries with heterosexual contact as primary mode of HIV transmission				Non-Sub-Saharan African countries with heterosexual contact as primary mode of HIV transmission				Non-Sub-Saharan African countrieswith homosexual contact or injection-drug use as primary mode of HIV transmission			
			
	No. of countries	Regression coefficient	p-value	*R*^2^	No. of countries	Regression coefficient	p-value	*R*^2^	No. of countries	Regression coefficient	p-value	*R*^2^
**Unadjusted regression model**	**35**			**0.68**	**24**			**0.70**	**32**			**0.49**
Percent of population Muslim		-0.013	0.023			0.031	0.02			0.021	0.51	
Percent of population Christian		0.0015	0.84			0.021	0.04			0.017	0.003	
Male circumcision prevalence^2^		-0.61	<0.001			-2.19	<0.001			-1.26	0.35	
												
**Adjusted regression model**^3^	**27**			**0.85**	**22**			**0.72**	**23**			**0.73**
Percent of population Muslim		-0.011	0.026			0.014	0.19			-0.036	0.26	
Percent of population Christian		-0.0044	0.48			0.0011	0.94			0.015	0.15	
Male circumcision prevalence^2^		-0.82	0.001			-1.60	0.001			0.47	0.70	

^1 ^Analyses conducted with natural log of HIV seroprevalence.

^2 ^Male circumcision prevalence was coded as 1 = low (<20%), 2 = intermediate (20–80%), and 3 = high (>80%).

^3 ^Model adjusted by country-specific measures including years since HIV was first reported, major geographical region, percent of population younger than age 25, percent of female adult illiteracy rate, percent of children fully immunized for diphtheria, tetanus, and pertussis, and number of doctors per 100,000 people.

Among all non-sub-Saharan African countries, male circumcision remained independently associated with HIV prevalence among countries in regions with heterosexual contact as the primary mode of HIV transmission, but not among countries in regions with either homosexual contact or injection drug use as the primary mode of HIV transmission (Table 4). When stratifying the countries by religious groups among countries with primarily heterosexual HIV transmission, each categorical increase of male circumcision prevalence was associated with a 8.94-fold (95% CI 4.30-18.60) decrease in adult HIV prevalence. In a multivariate model adjusted for 6 additional country-specific variables [5], each categorical increase of male circumcision prevalence was associated with a 4.94-fold (95% CI 2.29-10.65, p = 0.001) decrease in adult HIV prevalence. In a separate multivariate model, after stratifying the countries by religious groups among countries with primarily homosexual contact or injection drug use HIV transmission, male circumcision was not significantly associated with HIV prevalence (p = 0.35). When adjusting the multivariate model for the 6 additional country-specific variables [5], male circumcision prevalence was not significantly associated with HIV prevalence (p = 0.70).

## Discussion

This ecological study of 118 developing countries expands results from our previous analyses [5,22] by elucidating patterns and associations between male circumcision, religion, and infectious diseases, particularly HIV. Male circumcision, which is routinely practiced in the Middle East, northern and western Africa, and western Asia, was associated with lower rates of certain STIs, HIV and cervical cancer (a proxy for HPV), but not with infections transmitted by non-sexual routes. In general, more male circumcision was strongly associated with lower cervical cancer rates and fewer HIV cases, independent of religion. Furthermore, male circumcision was independently associated with HIV among countries with primarily heterosexual HIV transmission, and not among countries with primarily homosexual or injection drug use HIV transmission. These findings all suggest that male circumcision is a true protective factor that reduces the sexual transmission of HIV and possibly HPV, independent of Muslim and Christian religions.

An ecologic study of this type has limitations, as we have previously acknowledged [5,22]. In brief, ecological analyses cannot measure correlates of risk at the individual-level. Second, the temporal sequence of events in individuals is undetermined. Since the approximate age at circumcision varies by country, the results could be affected if a significant proportion delayed male circumcision until after initiation of sexual activity. Third, the validity of country-level data undoubtedly varies, and some data, such as HSV-2 prevalence, were not available for many countries. Although mean HSV-2 prevalence was lower among countries with more male circumcision, the limited number of countries with HSV-2 data (23 countries) gave relatively low power for examining the significance of this association. Fourth, and perhaps most importantly, statistics on the distribution and variation within countries were not complete. Some countries, such as Kenya, have widely varying regional prevalences of male circumcision and HIV [32]. For example, western Kenya, where less than 20% of males are circumcised, has a much higher HIV prevalence than regions of the country where nearly all men are circumcised [3,32]. The 2003 Kenya survey also found uncircumcised males had over three-fold higher HIV prevalence as compared to circumcised males, and HIV prevalence in circumcised men was nearly identical among the major religious groups (2.6% among Catholics, 3.0% in Protestant/other Christians, and 2.9% in Muslims) [32]. Finally, not all population-level measures that may impact infectious disease transmission, such as patterns of risk behaviors, condom availability and utilization, and injection drug use, were included in this analysis. Despite these limitations, findings from this ecological analysis support a biological relationship between male circumcision and certain STIs.

Previous studies, including a recent Cochrane review [16], have also found male circumcision to be associated with a reduced risk of HPV detection in men [14], cervical cancer in female partners [13], and HIV infection [3,11,12,15,17], while associations between male circumcision and HSV-2 and gonorrhea are less clear [15,19,43]. However, these studies were generally conducted among geographically-limited populations and many were unable to adequately control for religion. By comparison, our study described the global distribution of male circumcision prevalence, examined a large number of geographically-diverse countries and several infectious diseases, separately analyzed countries by primarily heterosexual and non-heterosexual transmission of HIV, and included relatively complete and recent surveillance data. Our study supports results of other studies by demonstrating independent associations of male circumcision with reduced cervical cancer rates and HIV cases, after stratifying the countries for Muslim and Christian religions, among a large sample of developing countries.

Others have described biologically plausible mechanisms by which male circumcision may reduce the transmission of certain STIs [18,19,44,45]. First, circumcised males may have less difficulty maintaining penile hygiene, which may reduce the acquisition of STIs by decreasing inflammation and the carriage time of pathogens in the foreskin [19,45]. Secondly, the non-keratinized epidermis of the prepuce in uncircumcised males may provide an easier portal of entry to STIs [19,44,45]. Third, the inner mucosal surface of the prepuce, which has a high density of HIV target cells (CD4+ T-cells, Langerhans cells, macrophages), has been shown to become more easily infected with HIV as compared to outer foreskin tissue [45]. Given these biological differences, it is entirely plausible that uncircumcised males are at a greater risk of acquiring some STIs, and of transmitting them to their sexual partners [12].

According to UNAIDS, heterosexual transmission was the primary mode of HIV transmission in Africa, the Middle East, the Caribbean, and South and South-East Asia, and homosexual transmission was the primary mode of HIV transmission in Latin America [37]. In our analysis, male circumcision was strongly associated with HIV among developing countries with heterosexual contact as the primary mode of HIV transmission, and not among developing countries whose primary mode of HIV transmission was not heterosexual contact (Eastern Europe, Central and Eastern Asia, Latin America, and the Pacific). These results further support the biological role of male circumcision as a protective factor in HIV transmission. Furthermore, the fact that HIV prevalence in many predominantly Christian countries that practice male circumcision, such as the Philippines, Benin, Ghana, Equatorial Guinea, and Gabon, is similarly low as in predominantly Muslim countries in the same regions, suggests that the biological effect of male circumcision may be at least as important as religion in determining HIV prevalence [24].

## Conclusion

This study demonstrates that male circumcision was strongly associated with lower cervical cancer rates and fewer HIV cases among countries with heterosexual contact as the primary mode of HIV transmission, independent of religion. One randomized controlled trial has demonstrated that male circumcision is highly protective of HIV acquisition [21]. Therefore, while HIV and cervical cancer are impacted by a complex set of biological, social, and public health influences, male circumcision appears to play a prominent role in decreasing transmission of certain STIs. Although male circumcision must not substitute for other HIV and STI prevention strategies [46], the international public health and medical community should consider the implications and practicalities of integrating safe, voluntary male circumcision services with existing HIV prevention programs, particularly in countries with low prevalence of male circumcision and high prevalence of sexually-transmitted HIV.

## Abbreviations

HIV – human immunodeficiency virus; HSV-2 – herpes simplex virus type-2; STIs – sexually-transmitted infections; UNDP – United Nations Development Programme; UNAIDS – The Joint United Nations Programme on HIV/AIDS; HPV – human papillomavirus;

## Competing interests

The author(s) declare that they have no competing interests.

## Authors' contributions

PKD, DTH, JPH, and RCB designed the study. PKD, DTH, and RCB collected and analyzed data. PKD, DTH, JPH, JDK, and RCB interpreted the results. PKD primarily wrote the manuscript. DTH, JPH, JDK, and RCB provided valuable insight for revising the manuscript. All authors read and approved the final manuscript.

## Pre-publication history

The pre-publication history for this paper can be accessed here:


